# Cold Stress Resistance of Tomato (*Solanum lycopersicum*) Seedlings Is Enhanced by Light Supplementation From Underneath the Canopy

**DOI:** 10.3389/fpls.2022.831314

**Published:** 2022-04-12

**Authors:** Tao Lu, Yangfan Song, Hongjun Yu, Qiang Li, Jingcheng Xu, Yong Qin, Guanhua Zhang, Yuhong Liu, Weijie Jiang

**Affiliations:** ^1^Institute of Vegetables and Flowers, Chinese Academy of Agricultural Sciences, Beijing, China; ^2^College of Horticulture, Xinjiang Agricultural University, Ürümqi, China; ^3^Natural Resources Bureau of Hutubi County in Xinjiang Province, Changji, China; ^4^Taizhou Academy of Agricultural Sciences, Taizhou, China; ^5^Agriculture and Animal Husbandry Comprehensive Inspection and Testing Center of Chifeng, Chifeng, China; ^6^Tibet Academy of Agriculture and Animal Husbandry Sciences Vegetable Research Institute, Lhasa, China

**Keywords:** photosynthetic efficiency, light responsiveness, stomatal traits, antioxidant enzyme, abiotic stress

## Abstract

Adverse environmental conditions, such as low temperature (LT), greatly limit the growth and production of tomato. Recently, light-emitting diodes (LEDs) with specific spectra have been increasingly used in horticultural production facilities. The chosen spectrum can affect plant growth, development, and resistance, but the physiological regulatory mechanisms are largely unknown. In this study, we investigated the effects of LED light supplementation (W:B = 2:1, light intensity of 100 μmol⋅m^–2^⋅s^–1^, for 4 h/day from 9:00 to 13:00) from above and below the canopy on tomato resistance under sub-LT stress (15/8°C). The results showed that supplemental lighting from underneath the canopy (USL) promoted the growth of tomato seedlings, as the plant height, stem diameter, root activity, and plant biomass were significantly higher than those under LT. The activity of the photochemical reaction center was enhanced because of the increase in the maximal photochemical efficiency (F_*v*_/F_*m*_) and photochemical quenching (qP), which distributed more photosynthetic energy to the photochemical reactions and promoted photosynthetic performance [the maximum net photosynthetic rate (*Pmax*) was improved]. USL also advanced the degree of stomatal opening, thus facilitating carbon assimilation under LT. Additionally, the relative conductivity (RC) and malondialdehyde (MDA) content were decreased, while the soluble protein content and superoxide dismutase (SOD) activity were increased with the application of USL under LT, thereby causing a reduction in membrane lipid peroxidation and alleviation of stress damage. These results suggest that light supplementation from underneath the canopy improves the cold resistance of tomato seedlings mainly by alleviating the degree of photoinhibition on photosystems, improving the activity of the photochemical reaction center, and enhancing the activities of antioxidant enzymes, thereby promoting the growth and stress resistance of tomato plants.

## Introduction

Under natural conditions, plants often encounter various stresses, including biotic and abiotic stresses, which impede plant growth and development and have adverse impacts on quality and productivity (Domenico et al., 2013; Zhou et al., 2020). As one of the main determinants of plant propagation and production, low temperature (LT) often occurs during late autumn, winter, and early spring in northern China (Shi et al., 2016; Nievola et al., 2017), causing a series of molecular, physiological, biochemical, and morphological changes to occur in plants (Khan et al., 2019). Previous studies have reported that cold stress reduces the net photosynthesis rate and maximal efficiency of photosystem (PS) II photochemistry (Devacht et al., 2011; Kalisz et al., 2016), increases cell relative electrical conductivity (Kim and Tai, 2011), increases the accumulation of soluble sugars that originate from starch metabolism (Lin et al., 2019), and promotes the activity of superoxide dismutase and catalase (Petrić et al., 2013). Several photoreceptors, such as phytochromes (phy) and cryptochromes (cry), have developed in plants to sense changing environments. Phy A is the predominant photoreceptor of far-red (FR) light and phy B is the primary photoreceptor of red (R) light (Chen and Chory, 2011). In addition, the transcription factor ELONGATED HYPOCOTYL5 (HY5) can be activated by photoreceptors to promote downstream photomorphogenesis (Li et al., 2021). Many studies have shown that the above molecules play key roles in cold tolerance (Chen and Chory, 2011; Li et al., 2021; Wang et al., 2021). It has been shown that light signals regulate chloroplast avoidance movement through phy to reduce photodamage in plants (Kasahara et al., 2002; Jaedicke et al., 2012; Suetsugu et al., 2017). Wang et al. (2018) found that phy is involved in photoprotection through the PROTON GRADIENT REGULATION5 (PGR5)-dependent cyclic electron flow pathway during cold stress and they suggested that phy A and phy B function antagonistically to regulate cold tolerance via abscisic acid-dependent jasmonate signaling (Wang et al., 2016). SIFHY3 and SIHY5 act together to enhance cold tolerance through the integration of myoinositol and light signaling in tomato (Wang et al., 2021). Bu et al. (2021) characterized 31 *B-BOX* (*BBX*) genes in tomato that play important roles in the plant response to cold and light signaling. Plants must maintain membrane fluidity at the cellular level in progressively cold and oxidized environments to overcome cold stress. As membranes are sensitive to damage, improved cold resistance helps to maintain membrane stability and, thus, minimize electrolyte leakage (Raju et al., 2018). In addition, reactive oxygen species (ROS), calcium (Ca^2+^), and plant hormones such as abscisic acid, brassinosteroids, and strigolactone all play key roles in plant cold tolerance (Demidchik et al., 2018; Khan et al., 2019; Lu et al., 2019; Cao et al., 2021). Hydrogen peroxide (H_2_O_2_) is the most stable ROS and previous studies have revealed that elevated levels of apoplastic H_2_O_2_ and increased respiratory burst oxidase homolog (RBOH)-encoded NADPH oxidase activity are related to acclimation-induced cross-tolerance (Zhou et al., 2014). Recently, the glutamate receptor-like (GLR) genes such as *GLR3.3* and *GLR3.5* were shown to mediate chilling tolerance by regulating apoplastic H_2_O_2_ production and redox homeostasis (Li et al., 2019). These various pathways work together to alter cold resistance.

As an energy source and signaling factor, light affects photosynthesis through complex and diverse photosensitive systems and regulates the structure and permeability of the membrane system, thereby changing the structure of cells and ultimately affecting their growth and metabolism (Molina et al., 1997; Grieco et al., 2012). In plant cultivation and production, metal-halide lamps and high-pressure sodium lamps are generally used to extend light duration or increase light intensity. However, these light sources also provide wavelengths that cannot be utilized efficiently or may not support photosynthesis and plant growth at all (Olive et al., 2013). Besides, one another disadvantage of these artificial lights is the reduction of light intensity with increasing the distance between lamps with leaves (Poorter et al., 2012). The positions of leaves at the top of the canopy vary as the plants increase in size. To maintain constant light intensity at the top of the canopy, the height of the lamp needs to be adjusted constantly; however, light at the bottom of the canopy is inevitably reduced (Rowse et al., 2016). These light sources also produce heat that is conducive to crop growth, but as thermal light sources, they cannot be placed very close to the plant surface or they will easily burn young tissues and cause leaf photoinhibition (Niinemets and Keenan, 2012; Li et al., 2021). In comparison, light-emitting diodes (LEDs) are considered to be a suitable light source for interlighting because of their low heat production (less likely to burn leaves), non-residual and non-toxic effects, and long operating lifetimes. In addition, LED lighting offers a specific monochromatic spectrum, thus favoring photomorphogenic responses such as the morphology and metabolite content of the leaves (Taulavuori et al., 2017). Commercial LED lamps typically combine blue and red wavelengths, as these wavelengths are highly absorbed by chlorophyll and, thus, promote photosynthesis and biomass production (Okamoto et al., 1996).

Improving the distribution of light in the canopy can improve the utilization efficiency of light and, thus, improve canopy photosynthesis. In the plant canopy, leaves at the top of the canopy usually absorb more light energy than it is necessary and the excess light energy is dissipated as heat and may result in photoinhibition. However, leaves at the bottom of the canopy usually have limited available light, which can also lead to photoinhibition (Keren and Krieger, 2011; Huang et al., 2018; Hikosaka, 2021). To improve the light use efficiency of the canopy, a variety of schemes have been proposed (Zhu et al., 2010; Long et al., 2015). Among these schemes, supplementary light from underneath the canopy has been proposed as a viable option. A comparison of light supplies placed above, inside, and underneath the canopy showed that light above the canopy only increased the light intensity of the plant tip, while the other two light positions improved the light distribution in the middle and bottom parts of the tomato plant; this was especially true for light supplied underneath the canopy, which made the whole light environment of the plant more uniform (Shao, 2019). Improving the distribution of light inside the canopy can increase light use efficiency and, hence, increase canopy photosynthesis. Several studies have shown that in the case of limited sunshine, supplemental lighting above or within the canopy promoted the growth of tomato plants and shortened the flowering time, thus increasing yield and economic efficiency (Na et al., 2012). Moreover, researchers found that supplemental light within the cowpea canopy delayed the senescence of interior leaves (Frantz et al., 2000). Additionally, supplying upward lighting from underneath retarded the senescence of outer leaves of lettuce and improved plant growth (Zhang et al., 2015). Therefore, lighting different parts of the plant canopy can be beneficial.

Seedlings cultivated under supplementary light are robust and have good resistance to adversity; moreover, the fruit quality of these plants is improved at the harvest stage (Lu et al., 2012). Studies also show that LED lighting application increases the resistance of strawberry to *Botrytis cinerea* and cucumber to root knot nematodes; it can also increase the stress resistance of gourd seedlings and pomegranate saplings (Meng et al., 2018; Khan and Siddiqui, 2021). Hence, using light manipulation to improve seedling resistance is regarded as a green energy technology. Tomato is the second most important vegetable crop grown in protected facilities worldwide and it has been reported that temperatures below sub-LT (15°C) must be avoided with most cultivars (Dominguez et al., 2005). It is necessary to enhance the cold resistance of tomato plants to minimize economic losses from low-temperature injury. However, to the best of our knowledge, no information is available about LED light application on the growth and development of tomato plants under LT. Our objective was to investigate how supplemental LED from underneath the canopy improves the resistance of tomato seedlings under sub-LT stress.

## Materials and Methods

### Plant Material and Growth Conditions

Tomato (*Solanum lycopersicum* “Moneymaker”) seeds were soaked in 55°C water for 30 min and pregerminated in a 28°C thermostat incubator. The germinated seeds were then sown in 72-cell trays filled with vermiculite. Seedlings at the two-leaf stage were cultivated in 15 cm × 13 cm pots with regular cultivation management and irrigated with half-Hoagland’s nutrient solution in a glasshouse. Seedlings at the six-leaf stage were separated into the five groups of 45 pots each and transferred to a phytotron (plant growth sodium lamps were used as the light source with approximately 300 μmol⋅m^–2^⋅s^–1^) for 3 days to adapt to the following environment: a relative humidity of 60%, a photoperiod of 12 h (7:00–19:00), and a 25/15°C (day/night) air temperature.

### Supplemental Lighting and Sub-Low Temperature Treatments

Light-emitting diode lighting systems (Philips, Eindhoven, Netherlands) were applied as supplemental light sources. The polychromatic light was combined white and blue light (W:B = 2:1) with a photosynthetic photon flux density (PPFD) of 100 μmol⋅m^–2^⋅s^–1^ measured at 10 cm from the LED module. Seedlings were divided into different phytotrons for the following treatments: CK, seedlings under natural temperature (25/15°C); CK + USL, seedlings under natural temperature with supplemental lighting from underneath the canopy; LT, seedlings under sub-LT (15/8°C); LT + USL, seedlings under LT with supplemental lighting from underneath the canopy; and LT + TSL, seedlings under LT with supplemental lighting from above the canopy. Light was provided from 9:00 to 13:00. The fifth fully expanded leaves and roots were collected for physiological and biochemical analysis.

### Measurement of Gas Exchange and Chlorophyll Fluorescence

The gas exchange, chlorophyll fluorescence, and P700 redox state were measured *in vivo* by using the LI-6400XT Photosynthesis System (Li-Cor Incorporation, United States) and the Dual PAM-100F (Heinz Walz, Effeltrich, Germany) as described in previous reports (Grieco et al., 2012; Pietrzykowska et al., 2014). The light-adapted curves were recorded after 2 min of exposure to various PPFDs (Lu et al., 2019; Li et al., 2021).

### Determination of Plant Growth and Root Morphology

Plant growth was evaluated by measuring plant height, stem diameter, and wet and dry weight. Root morphology was scanned using an “Epson Perfection V168” photo flatbed scanner (Epson, Long Beach, United States) and root activity was measured with the triphenyltetrazolium chloride (TTC) method (Ou et al., 2011).

### Observation of Leaf Stomatal Microstructure

To observe the microstructure of the stomata, the torn leaf epidermis was immersed in a transparent nail polish buffer and sectioned onto slides for microimaging. Images of each strip were taken under a Leica microscope (Leica Microsystems AG, Solms, Germany) equipped with a Nikon NIS-F1 CCD camera and a Nikon DS-U3 controller (Nikon, Tokyo, Japan). Enumeration and measurement of stomatal parameters were conducted with 20 and 100× objective lenses (Lu et al., 2017a).

### Analysis of Chlorophyll, Malondialdehyde, and Soluble Protein Content

The chlorophyll content was measured with the lixiviating method (Muneer et al., 2020). The contents of Malondialdehyde (MDA) and soluble protein were measured based on the thiobarbituric acid (TBA) assay and Bradford method, respectively (Chang et al., 2016).

### Estimation of Relative Conductivity and Cell Damage Rate

The estimations of Relative Conductivity (RC) and cell damage rate were carried out according to Yang et al. (1996) report. Fresh leaf samples were washed and cut into 1 cm strips. Leaves (0.1 g) were soaked in 20 ml deionized water for 12 h at room temperature (RT) and the initial conductivity was measured as R1. Then, leaves were heated in boiling water for 30 min and cooled to RT. After shaking, the conductivity was measured as R2. RC = R1/R2 × 100%. Cell damage rate = [1-(R1/R2)/1-(C1/C2)] × 100%. C1 and C2 are the conductivities of the blank controls.

### Assessment of Antioxidant Enzyme Activity

The activities of superoxide dismutase (SOD), peroxidase (POD), and catalase (CAT) were measured with plant physiology kits (Jiancheng Biotechnology Corporation Ltd., Nanjing, China). Half gram of fresh leaves were ground into a fine powder with liquid nitrogen and extracted with ice-cold 50 mM phosphate buffer (pH 7.8). The extracts were centrifuged at 4°C and 10,000 × g for 15min and the supernatants were used to evaluate the enzyme activity based on the enzyme assay with a Multiskan Sky Visible Spectrophotometer (Thermo Fisher Scientific, Massachusetts, United States) (Zhao et al., 2017).

### Statistical Analysis and Visualization

Five treatments were setup in this experiment with three replicates for each treatment. Related indicators were measured for three separate plants for each replication. The data were the mean ± *SD* of three replicates. Values were compared between the five treatments with Duncan’s multiple comparison test at a probability level of 0.05 in SPSS version 20 software (SPSS Incorporation, IBM Armonk, New York, United States). Figures were drawn with GraphPad Prism version 6.01 (GraphPad Software Incorporation, La Jolla, United States).

## Results

### Supplemental Lighting From Underneath Promotes the Growth and Development of Tomato Seedlings Under Low Temperature Stress

**FIGURE 1:**
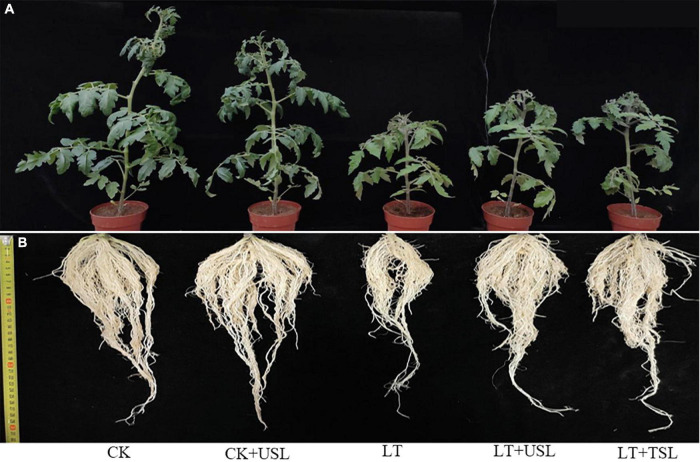
Phenotypic observation of the aboveground **(A)** and underground **(B)** morphology of tomato seedlings under low temperature (LT) stress with light supplementation from underneath the canopy. CK, seedlings under natural temperature; CK + USL, seedlings under natural temperature with supplemental lighting from underneath the canopy; LT, seedlings under sub-LT; LT + USL, seedlings under sub-LT with supplemental lighting from underneath the canopy; and LT + TSL, seedlings under sub-LT with supplemental lighting from above the canopy.

Supplemental lighting from underneath (USL) promoted the growth of aboveground and underground parts of tomato seedlings and improved their morphological structure under sub-LT stress (Figures 1A,B). Compared with the CK, the plant heights of CK + USL, LT, LT + USL, and LT + TSL plants were significantly decreased. LT + USL and LT + TSL effectively increased plant height by 27 and 24% compared to LT, respectively, and there was no significant difference between them. In addition, root length was significantly increased by supplementary light. The effect of LT + USL was better than that of LT + TSL, as both produced longer roots than LT by 26 and 12%, respectively, and there was a significant difference between these treatments. Other growth indices, such as stem and root diameter, as well as root activity, showed the same trend: LT resulted in the diameter of stems and roots becoming thinner. After supplemental lighting, both the indices became larger (as shown in Table 1). Additionally, plant biomass was significantly decreased by LT and the dry weights of roots, stems, and leaves were lower than those under CK by 34, 50, and 36%, respectively. When seedlings were given USL, these weights were improved by 17, 19, and 29%, which were significantly higher than those under LT. Supplemental lighting from above the canopy had a similar, but weaker improvement effect.

**TABLE 1:** Effects of USL on the plant and root growth indices, biomass allocation, and root activity of tomato seedlings under low temperature (LT).

			Dry weight						
Treatment	Plant height	Stem diameter	Leaf	Stem	Root	Total	Root activity	Total root length	Average root diameter mm
	cm	mm	g	g	g	g	mg⋅g⋅h^–1^	m	
CK	65 ± 2^a^	8.0 ± 0.1^b^	6.2 ± 0.2^b^	3.2 ± 0.1^b^	1.1 ± 0.1^bc^	10.5 ± 0.5^b^	1.50 ± 0.03^b^	13.5 ± 0.5^b^	0.42 ± 0.02^b^
CK + USL	56 ± 4^b^	8.4 ± 0.1^a^	6.9 ± 0.3^a^	3.6 ± 0.1^a^	1.4 ± 0.1^a^	11.8 ± 0.4^a^	1.65 ± 0.02^a^	16.2 ± 0.9^a^	0.46 ± 0.02^a^
LT	33 ± 3^d^	7.5 ± 0.1^d^	4.1 ± 0.2^d^	1.6 ± 0.1^d^	0.7 ± 0.1^d^	6.4 ± 0.3^d^	1.20 ± 0.01^d^	8.6 ± 0.2^e^	0.33 ± 0.01^d^
LT + USL	42 ± 4^c^	7.8 ± 0.1^c^	4.8 ± 0.2^c^	1.9 ± 0.1^c^	0.9 ± 0.1^c^	7.6 ± 0.3^c^	1.30 ± 0.01^c^	10.8 ± 0.3^c^	0.37 ± 0.01^c^
LT + TSL	41 ± 4^c^	7.8 ± 0.1^c^	4.5 ± 0.1^d^	1.8 ± 0.1^c^	0.8 ± 0.1^c^	7.1 ± 0.2^c^	1.29 ± 0.02^c^	9.6 ± 0.4^d^	0.36 ± 0.01^c^

*Data represent the means ± SE (n = 9). Different superscript letters in the same column indicate significant difference (P < 0.05), and the same letter indicates no significant difference (P > 0.05).*

### Supplemental Lighting From Underneath Improves Leaf Photosynthetic Capacity Under Low Temperature Stress

As shown in Table 2, the contents of Chl *a*, Chl *b*, and total chlorophyll were significantly increased by CK + USL. LT caused the above contents to decrease by 21, 32, and 23% and Chl *a*/Chl *b* to decrease by 17%. Compared with LT, LT + USL significantly increased the contents of Chl *a*, total chlorophyll, and Chl *a*/Chl *b* by 9, 5, and 29%, respectively; however, the Chl *b* content was decreased by 16%.

**TABLE 2:** Effects of USL on the leaf chlorophyll content of tomato seedlings under LT stress.

	Chl *a* content	Chl *b* content	Total chlorophyll content	
		
Treatment	mg⋅g^–1^FW	mg⋅g^–1^FW	mg⋅g^–1^FW	Ratio of Ch*a*/Ch*b*
CK	1.63 ± 0.01^b^	0.38 ± 0.02^b^	2.01 ± 0.02^b^	4.30 ± 0.29^d^
CK + USL	1.72 ± 0.04^a^	0.33 ± 0.02^a^	2.05 ± 0.03^a^	5.24 ± 0.22^b^
LT	1.29 ± 0.02^e^	0.26 ± 0.02^c^	1.55 ± 0.01^d^	4.95 ± 0.34^c^
LT + USL	1.41 ± 0.04^c^	0.22 ± 0.01^d^	1.63 ± 0.03^c^	6.41 ± 0.45^a^
LT + TSL	1.34 ± 0.02^d^	0.23 ± 0.01^d^	1.57 ± 0.02^cd^	5.83 ± 0.33^a^

*Different superscript letters in the same column indicate significant difference (P < 0.05), and the same letter indicates no significant difference (P > 0.05).*

**FIGURE 2:**
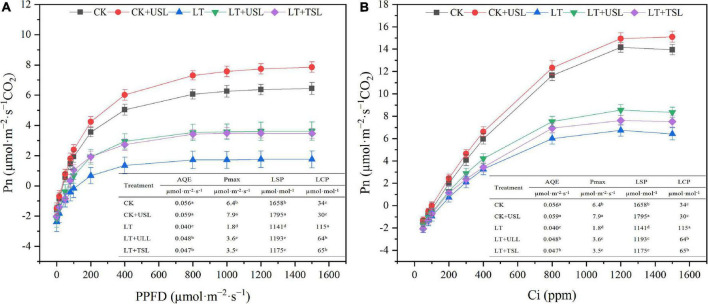
Effects of USL on the Pn-light response curve **(A)** and Pn-CO_2_ response curve **(B)** of tomato leaves under LT. *AQE*, apparent quantum efficiency; *LSP*, light saturation point; *LCP*, light compensation point; *CE*, carboxylation efficiency; *CSP*, CO_2_ saturation point; *CCP*, CO_2_ compensation point; *Pmax*, maximum net photosynthetic rate.

The chlorophyll content affects photosynthesis and light supplementation significantly increased the apparent quantum efficiency (*AQE*) and light saturation point (*LSP*). Compared with LT, the *AQE* and *LSP* under LT + USL were significantly increased by 20 and 6%, respectively, while the light compensation point (*LCP*) was significantly decreased by 76% (Figure 2A). In addition, the carboxylation efficiency (*CE*) and CO_2_ saturation point (*CSP*) significantly increased by 15 and 4%, respectively, while the CO_2_ compensation point (*CCP*) significantly decreased by 11% (Figure 2B). Additionally, the maximum net photosynthetic rate (*Pmax*) of the Pn-light and Pn-CO_2_ response curves were both improved by USL under LT stress.

### Supplemental Lighting From the Underneath Relieves the Photoinhibition Degree and Enhances the Energy Distribution in Photosystem II and Photosystem I Under Low Temperature Stress

**FIGURE 3:**
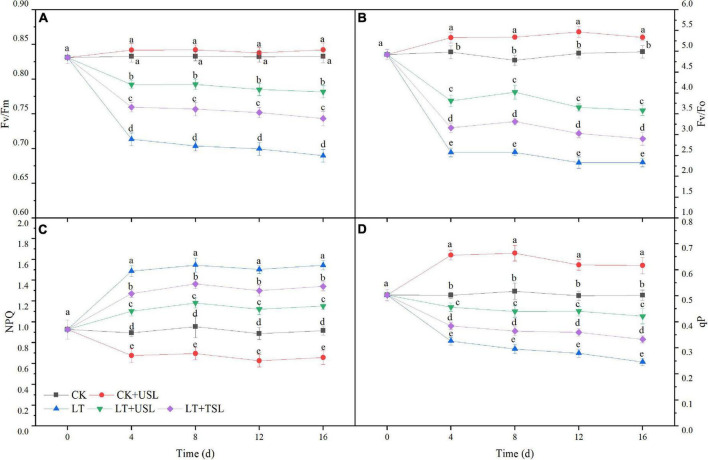
Effects of USL on the PS II reaction center of tomato leaves under LT stress. **(A)** F_*v*_/F_*m*_, the maximal photochemical efficiency of PS II; **(B)** F_*v*_/F_0_, potential photochemical activity of PS II; **(C)** NPQ, light-induced non-photochemical quenching; and **(D)** qP, photochemical quenching coefficient.

Photoinhibition occurred in tomato leaves under LT stress. As shown in Figures 3A,B,D LT caused a significant decrease in the maximal photochemical efficiency of PS II (F_*v*_/F_*m*_), the potential photochemical activity of PS II (Fv/Fo), and photochemical quenching (qP); however, USL significantly increased these values by 78, 54, and 71%, respectively. In addition, non-photochemical quenching (NPQ) of LT was increased by 68%, which was significantly higher than that of the CK, while LT + USL significantly decreased NPQ by 25% (Figure 3C). Thus, USL could effectively alleviate the PS II photoinhibition in tomato leaves caused by LT stress and the activity of the PS II reaction center was greatly improved.

**FIGURE 4:**
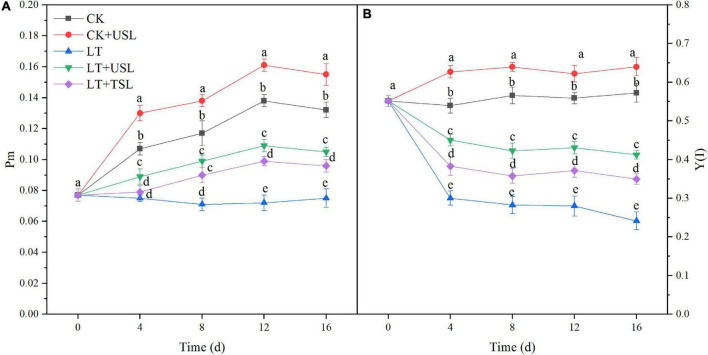
Effects of USL on the PS I reaction center of tomato seedlings under LT stress. **(A)** Pm, the maximal P700 changes and **(B)** Y(I), the effective quantum yield of PS I.

The maximal P700 changes (Pm) as well as the effective quantum yield of PS I [Y(I)] decreased from day 4 after LT stress and the decrease in the amplitude increased with prolonged stress duration. Hence, PS I activity was inhibited. Compared with LT, at day 16, LT + USL significantly increased the values of Pm and Y(I) by 43 and 54%, respectively (Figure 4). Therefore, USL is good for tomato PS I.

**FIGURE 5:**
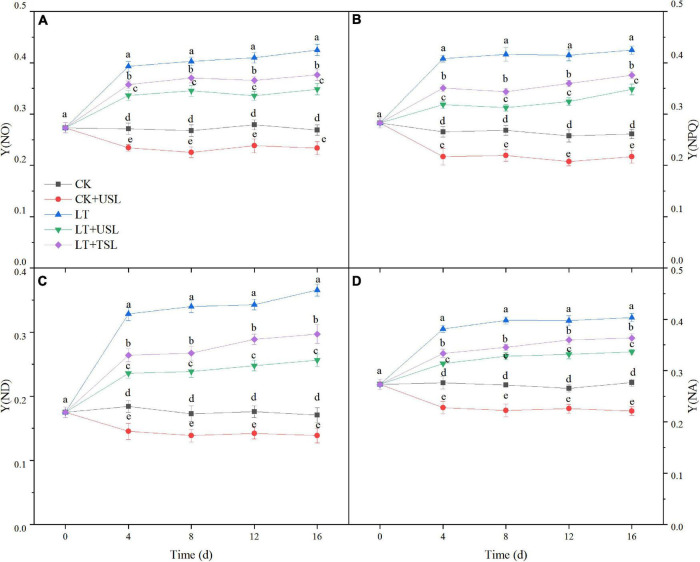
Effects of USL on the energy fluxes between photosystems in tomato leaves under LT stress. **(A)** Y(NO), the quantum yield of non-regulated energy dissipation; **(B)** Y(NPQ), the quantum yield of regulated energy dissipation; **(C)** Y(ND), PS I donor side limitation; and **(D)** Y(NA), PS I acceptor side limitation.

In this study, we measured the direct energy flow across both the PS II and PS I. As shown in Figure 5, the difference in quantum yields increased over time. Compared to the CK, the regulatory and non-regulatory quantum yields of energy dissipation [Y(NPQ) and Y(NO)] were both significantly increased by LT. Once USL was applied, Y(NPQ) and Y(NO) were both decreased significantly compared with LT (Figures 5A,B). The Y(I) of LT decreased gradually due to an increase in the acceptor-side limitation of PS I [Y(NA)] and an increase in the donor-side limitation of PS I [Y(ND)]. However, the Y(NA) and Y(ND) values of LT + USL were significantly lower than those of LT (Figures 5C,D). These results suggested that applying USL enhanced the energy fluxes between PS II and PS I.

### Effects of Supplemental Lighting From Underneath on Leaf Stomatal Density and Morphology Under Low Temperature Stress

**FIGURE 6:**
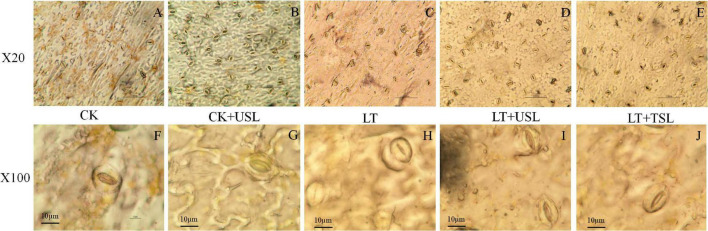
Effects of USL on the stomatal morphology of tomato leaves under LT stress. **(A–E,F–J)** are the stomatal morphology observed under 20 and 100X objective lenses, respectively.

Compared with the CK, LT decreased the density of stomata by 13% (Figures 6A,C and Table 3). Supplementation with light increased the density of stomata in the leaves; for example, the stomatal number of CK + USL was 36% higher than that of the CK (Figure 6B and Table 3). In addition, the stomatal numbers of LT + USL and LT + TSL were 41 and 16% higher than those of LT (Figures 6D,E and Table 3). In addition, USL effectively improved the stomatal aperture of tomato leaves under LT stress. By observing stomatal morphology and analyzing apparent characteristics, we found that stomatal area was significantly decreased by LT, but with USL or TSL, it was significantly elevated. The stomatal area of LT + USL was the largest because both the vertical diameter and transverse diameter were increased (Figures 6F,G and Table 3).

**TABLE 3:** Effects of USL on the characteristics of the tomato stomatal apparatus under LT stress.

Treatments	Vertical diameter	Transverse diameter	Area	Number
		
	μm	μm	μm^2^	
CK	16.48 ± 0.06^b^	5.14 ± 0.27^c^	66.54 ± 3.29^c^	30.33 ± 1.53^c^
CK + USL	19.72 ± 0.52^a^	9.56 ± 0.36^a^	148.03 ± 8.55^a^	41.33 ± 1.53^a^
LT	12.43 ± 0.72^c^	3.59 ± 0.12^d^	34.96 ± 0.97^d^	26.33 ± 1.53^d^
LT + USL	16.71 ± 0.23^b^	6.08 ± 0.45^b^	79.82 ± 6.18^b^	37.00 ± 2.00^b^
LT + TSL	16.72 ± 0.78^b^	4.99 ± 0.08^c^	65.48 ± 3.71^c^	30.67 ± 1.15^c^

*One slice was made for each plant, and nine slices were made for each treatment. The number of stoma in 3 non-adjacent visual fields in each slice was counted under a 20× objective lens. To measure stomatal vertical and transverse diameters, 6 complete and clear stomata were chosen for each slice under a 100× objective lens, with a total of 54 stomata for each treatment. Different superscript letters in the same column indicate significant difference (P < 0.05), and the same letter indicates no significant difference (P > 0.05).*

### Effects of Supplemental Lighting From Underneath on Membrane Lipid Peroxidation and Antioxidant Enzyme Activity Under Low Temperature Stress

**FIGURE 7:**
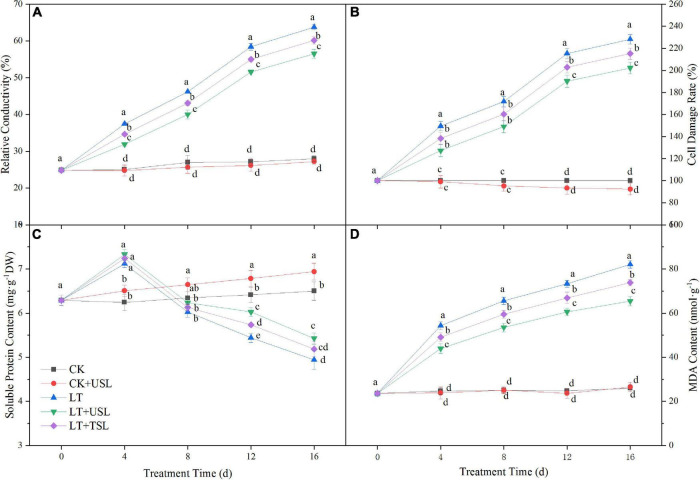
Effects of USL on the membrane lipid peroxidation of tomato seedlings under LT stress. **(A)** Relative conductivity and **(B)** cell damage rate reflect the status of plant membrane system; **(C)** Soluble protein content and **(D)** MDA content reflect the lipid peroxidation degree of plant membrane.

Stress conditions will increase the permeability of the cell membrane, leading to electrolyte extravasation in cells. In this study, LT gradually increased the RC with the extension of stress duration and the cell damage rate was seriously aggravated. Compared with the CK, these values were increased by 128 and 228%. However, supplemental lighting reduced the damage degree and the RC and cell damage rate of LT + USL were decreased by 13 and 11%, respectively (Figures 7A,B). Soluble protein is an important osmotic regulator in plants and the MDA content directly affects lipid peroxidation. In contrast to RC, the soluble protein content showed an initial increasing trend and then a decreasing trend under LT; after 16 days, this content had decreased by 23%. Compared with LT, the soluble protein content of LT + USL was significantly increased by 10%, while the MDA content was significantly decreased by 20%, indicating that USL alleviated the stress degree.

Plants rely on a variety of antioxidant enzymes, such as SOD, CAT, and POD, to remove ROS. As shown in Table 4, SOD activity was significantly increased and CAT activity was significantly decreased by LT. Compared with LT, the activities of both the SOD and CAT activities were significantly increased; however, the activity of POD showed no significant differences among the treatments, except CK + USL.

**TABLE 4:** Effects of USL on the activities of antioxidant enzymes in tomato leaves under LT stress.

	SOD	POD	CAT
	
Treatments	U⋅g^–1^FW⋅min^–1^	U⋅g^–1^FW⋅min^–1^	U⋅g^–1^FW⋅min^–1^
CK	123 ± 7^d^	215 ± 18^b^	315 ± 10^b^
CK + USL	161 ± 6^c^	365 ± 23^a^	377 ± 13^a^
LT	216 ± 12^b^	210 ± 23^b^	160 ± 7^d^
LT + USL	257 ± 11^a^	213 ± 25^b^	178 ± 8^c^
LT + TSL	237 ± 12^ab^	212 ± 28^b^	170 ± 10^cd^

*Different superscript letters in the same column indicate significant difference (P < 0.05), and the same letter indicates no significant difference (P > 0.05).*

## Discussion

Low temperature represents an important environmental factor affecting vegetable growth to a great extent. Under LT, plant height, stem diameter, and the growth of leaves of eggplant and tomato are inhibited (Cui et al., 2016; Shi et al., 2019). The key negative effect of chilling on cucumber and pepper is a reduction in biomass and photosynthetic capacity (Yong et al., 2003; Ikkonen et al., 2018). LT also induces chloroplast damage and affects photosynthetic physiological metabolism in thylakoid membranes (Shi et al., 2016; Yang et al., 2018), where the functions of sunlight capture, electron transmission, and energy conversion occur. Light is an energy and signaling factor that influences photosynthesis through complex plant photosystems and changes cell structure by regulating the permeability of biofilm systems, ultimately affecting plant growth and metabolism. To solve the shortage of sunlight in greenhouse cultivation and alleviate plant stress, artificial light supplementation technology has become one of the important ways to improve the production efficiency of facility agriculture. Thus, it is crucial to choose the best method of light supplementation and understand the physiological mechanism of stress resistance enhancement.

Stomatal characteristics are closely related to stomatal conductance and a higher stomatal conductance is always accompanied by greater photosynthesis (Zhang et al., 2019). Kim et al. (2004) found that the stomatal opening and Pn of *Chrysanthemum* tissue-cultured seedlings with red and blue mixed LEDs were largely enhanced. Previous studies in *Arabidopsis* also showed that the existence of blue light increased the number of stomata and stimulated stomatal opening (Yang et al., 2020). Under LT stress, USL not only increased the stomatal density, but also promoted stomatal opening by increasing the vertical and transverse diameters (Figure 6 and Table 3), which might partly explain the significant increase in *Pmax* observed in LT + USL plants. Moreover, USL may contributed to the activation of Rubisco (Wu et al., 2020), which can be reflected by the improved *CE* in LT + UTL seedlings (Figure 2B). Kinoshita et al. (2001) suggested that blue light promoted the absorption of the carotenoid zeaxanthin, thus promoting the opening of stomata. Li et al. (2020) believed that a blue LED light source directly promoted stomatal opening. Kang et al. (2009) and Yang et al. (2020)considered that stomatal opening was regulated by phy and cry. Although not definitive, most studies show that blue light can stimulate the expansion of stomatal opening and improve plant photosynthesis. Our physiological data also revealed that *Pmax*, *AQE*, *LSP*, *CE*, *CSP*, Chl *a*, and Chl *b* in tomato leaves were decreased by LT (Figures 2–4 and Table 2). However, USL significantly increased these parameters. As the main photosynthetic pigment, chlorophyll is capable of capturing, transmitting, and converting light energy (Jian et al., 2016). The reduction in chlorophyll content in tomato leaves by LT stress affected photosynthetic efficiency and aggravated photoinhibition (Table 2); however, LT + USL treatment effectively reduced the degradation of chlorophyll to improve leaf chlorophyll content and, thereby, maintain the high photosynthetic capacity of chloroplasts. LT + UTL-grown plants displayed lower LCP and CCP, which are characteristics that are conducive to the accumulation of organic matter and indicate stronger photosynthetic capacity (Cui et al., 2016; Rasmusson et al., 2020).

Photoinhibition is defined as a decrease in photosynthetic efficiency under strong light conditions, in which the photon input exceeds the requirements of photosynthesis (Barber and Andersson, 1992; Lu et al., 2017b). Photoinhibition may occur under other stresses as long as the light intensity and duration reach a certain photon threshold (Meng et al., 2017). In this study, we found that LT stress exacerbated the photoinhibition degree, as evidenced by a decrease in Fv/Fm, F_*v*_/F_0_, and Pm (Figures 3A, 4A), resulting in reduced light energy utilization (Sultana et al., 1999). USL significantly attenuated these parameters by increasing Y(I) and decreasing Y(NO) (Figures 4B, 5A). Recent studies suggest that moderate phosphorylation of LHC II and PS II makes PS I complexes move to the edge of the grana, which transfers sufficient excitation energy to PS I and alleviates the photoinhibition of PS II (Grieco et al., 2012; Pietrzykowska et al., 2014; Lima-Melo et al., 2019). Conversely, the photoinhibition of PS II within a controllable range can protect PS I from photoinhibition by preventing ROS production and regulating the electron transport chain (Takagi et al., 2016; Lima-Melo et al., 2019). According to Wang et al. (2020), LT destroys PQ and electron transport from PQH_2_ to PS I, which leads to an imbalance in electron consumption and light reactions, resulting in an increased degree of membrane lipid peroxidation and cell damage (Figures 7B,D). Fortunately, USL significantly decreased Y(NA), indicating that the PS I acceptor side limitation under LT was alleviated (Figure 5D). Recent studies suggest that this alleviation is due to the promotion of the NADP^+^/NADPH ratio and the number of available oxidized forms of NADP (Grieco et al., 2012; Lima-Melo et al., 2019; Wang et al., 2020). In this study, a large decrease in Pm and an increase in Y(ND) and Y(NO) under LT showed that the photoinhibition of PS I occurred rapidly upon the onset of an imbalance between the donor and acceptor side of PS I (Figures 4A, 5C,D). However, USL not only stimulated the photoprotection mechanism on the donor side, but also reduced the photodamage on the acceptor side to reduce PSI photoinhibition and enhance the Calvin cycle. Moreover, as the PS I activity cannot be restored to the control level, these results supported other findings suggesting that chloroplast antioxidant scavengers cannot prevent PS I photoinhibition in the case of donor/acceptor side imbalance (Takagi et al., 2016; Lima-Melo et al., 2019; Lu et al., 2020). Y(NPQ) and Y(NO) represent the activity and energy distribution of the PS II reaction center. In this study, they were both increased by USL under LT stress (Figures 5A,B), implying that LT + USL treatment increased the quantity of light absorbed by the reaction center and partially promoted PS II opening of tomato seedlings under LT stress (Klughammer and Schreiber, 2008). However, the excess light energy still could not dissipate through the regulatory mechanism of seedlings, which was reflected by the higher Y(NO) compared with the CK and the damage to the photosynthetic system was caused by LT stress. In addition, USL effectively diminished the Y(NO) proportion and enhanced the photochemical energy conversion, as Y(NPQ) remained higher than that under LT. These results suggested that the application of USL to plants under LT stress could enhance photosynthesis due to the enhancement of light harvesting efficiency caused by heightening of the response of the Mg branch through USL, which mainly increased the chlorophyll content (Wu et al., 2018).

Photosynthetic activity is highly affected by ROS; excess ROS production caused by disordered photosynthetic redox homeostasis will damage the cell membrane, leading to intracellular ion efflux (Lima-Melo et al., 2019). Under LT stress, ROS accumulation resulted in the peroxidation of cell membrane lipids, as reflected by the significant increase in the MDA content (Figure 7D) and the decrease in the soluble protein content (Figure 7C), which led to disruption of the physiological function of tomato plants and could even cause cell death (Figures 7A,B; Fahnenstich et al., 2008; Cao et al., 2021). The change in ion exosmosis and the level of cell damage can be reflected by electrolyte leakage measurements. The values of RC increased in stressed plants under LT; however, supplemental lighting significantly decreased this value and that of the cell damage degree rate (Figures 7A,B). Many studies use 50% electrolyte leakage as the critical survival threshold, although many plants die after more than 30% electrolyte leakage (Helena et al., 2017). A lower RC value below 50% was measured for USL compared to TSL. According to Helena et al. (2017) and Demidchik et al. (2018), the increase in the concentration of soluble protein, an osmotically active substance, by USL (Figure 7C) results in a decrease in the osmotic potential, which is a cold tolerance strategy that protects the structural integrity of cell membranes and proteins.

Plants have evolved many photoprotective mechanisms to reduce ROS formation and mitigate photooxidative damage (Fahnenstich et al., 2008). The increase in NPQ reflects the energy dissipation mechanism that protects the photosynthetic system by dissipating excess energy as heat and preventing oxidative damage (Jia et al., 2019). The decrease in qP suggests that the redox state of QA, which is a PS II primary electron receptor, is not good for electron transfer (Maxwell and Johnson, 2000). In this study, NPQ was decreased and qP was increased by LT + USL treatment throughout the entire LT stress duration (Figures 3C,D), indicating a decrease in the level of energy dissipation and an increase in the electron transfer activity. According to a previous study, the impairment of *SlBBX7*, *SlBBX9*, and *SlBBX20* suppresses the photosynthetic response and NPQ immediately after cold stress; thus, these genes positively regulate cold tolerance in tomato plants by preventing photoinhibition and enhancing photoprotection (Bu et al., 2021). The antioxidative mechanism is another important regulatory balance between the production and scavenging of ROS. Previous studies have shown that in stressed plants, the generated ROS induce antioxidant enzymes such as SOD, POD, and CAT to scavenge harmful compounds (Yu et al., 2016). These key enzymes work together to maintain the steady-state level of free radicals in plants and prevent the disorders of plant physiology and biochemistry caused by free radicals. Under cold stress, the high accumulation of H_2_O_2_ was accompanied by upregulation of Ca^2+^-dependent protein kinases (CPKs) (Lv et al., 2018) and was responsible for the activation of antioxidant systems, such as SOD, CAT, ascorbate peroxidase, phenols, and anthocyanins (Hajihashemi et al., 2020). In this study, higher SOD, CAT, and POD activities were observed in LT + USL-treated tomato plants than in LT-treated plants (Table 4), indicating that USL reduced LT-induced damage to the cell membrane of tomato leaves (Moura et al., 2018; Cao et al., 2021). Maintaining the integrity of membrane and organelle is closely related to ROS scavenging capacity and is considered to be a particular challenge under cold stress (Pennycooke et al., 2005; Nievola et al., 2017; Hajihashemi et al., 2020).

There is a balance and exchange between the plant defense response and plant growth promotion. Researchers have reported that plant height, stem diameter, and biomass production are external indicators of plant aboveground development (Kang and Kong, 2016). In this study, the lower shoot height, thinner stem diameter, and lighter shoot biomass of LT-treated plants (Figure 1A and Table 1) indicated that shoot growth was sensitive to sub-LT stress, which was consistent with the results of Helena et al. (2017). However, once tomato plants under LT were given USL, the leaves became larger, the chlorophyll content increased, and the photosynthetic activity increased, accordingly producing more photosynthetic products, which could promote the growth of plants (Zhang et al., 2017). The utilization of USL benefited not only shoot growth, but also root growth, which was clearly greater than that under LT (Figure 1B and Table 1), suggesting improved rooting. Generally, the growth of underground roots is closely related to the rhizosphere environment. After the application of USL to the aboveground leaves, the stress degree of LT was alleviated and the underground root absorption (especially nitrate nitrogen) was improved, which might promote the root growth of tomato seedlings, as confirmed by a large number of studies where nitrate nitrogen stimulated lateral root formation and increased root length (Jampeetong and Brix, 2009; Zhou et al., 2020).

## Conclusion

Cold resistance in plants is a multifaceted physiological trait. We present a way to effectively enhance the LT tolerance of tomato seedlings, i.e., supplemental lighting from underneath canopies. In line with physiological observations, the adaptation of tomato seedlings to sub-LT stress mainly depends on the enhancement of osmotic regulation, improvement of antioxidant enzyme activities, promotion of photosystem photochemical activities, and improvement of plant and root development. This study suggests a positive role for supplemental lighting from underneath the leaf canopy in protecting the plant against the hazards of cold stress. Moreover, the integration of light and temperature signals by plants to adapt to adverse stress remains to be further studied.

## Data Availability Statement

The original contributions presented in the study are included in the article/supplementary material, further inquiries can be directed to the corresponding author/s.

## Author Contributions

WJ, YQ, and HY made the study plan. YS, TL, QL, and JX performed the experiments. QL, HY, and YL collected the materials. TL, HY, GZ, YL, and WJ analyzed the data. TL, YS, GZ, and HY wrote the manuscript. All authors discussed the results and commented on the manuscript and gave final approval for publication.

## Conflict of Interest

The authors declare that the research was conducted in the absence of any commercial or financial relationships that could be construed as a potential conflict of interest.

## Publisher’s Note

All claims expressed in this article are solely those of the authors and do not necessarily represent those of their affiliated organizations, or those of the publisher, the editors and the reviewers. Any product that may be evaluated in this article, or claim that may be made by its manufacturer, is not guaranteed or endorsed by the publisher.
